# Effect of a fermented dietary supplement containing chromium and zinc on metabolic control in patients with type 2 diabetes: a randomized, placebo-controlled, double-blind cross-over study

**DOI:** 10.3402/fnr.v60.30298

**Published:** 2016-06-23

**Authors:** Yu-Mi Lee, Petra Wolf, Hans Hauner, Thomas Skurk

**Affiliations:** 1 ZIEL Institute for Food and Health, Clinical Nutritional Medicine, Technical University of Munich, Munich, Germany; 2 Institute for Medical Statistics and Epidemiology, Klinikum rechts der Isar, Technical University of Munich, Munich, Germany; 3 Institute of Nutritional Medicine, Klinikum rechts der Isar, Technical University of Munich, Munich, Germany; 4 ZIEL Institute for Food and Health, Core Facility Human Studies, Technical University of Munich, Munich, Germany

**Keywords:** intervention study, human, free-living condition, type 2 diabetes, HbA_1c_

## Abstract

**Background:**

For the increasing development of type 2 diabetes dietary habits play an important role. In this regard, dietary supplements are of growing interest to influence the progression of this disease.

**Objective:**

The aim of this study was to investigate the effect of a cascade-fermented dietary supplement based on fruits, nuts, and vegetables fortified with chromium and zinc on metabolic control in patients with type 2 diabetes mellitus.

**Methods:**

This was a randomized, placebo-controlled, double-blind, intervention study under free-living conditions using a cross-over design. Thirty-six patients with type 2 diabetes mellitus were enrolled and randomized either to receive a cascade-fermented dietary supplement enriched with chromium (100 µg/d) and zinc (15 mg/d) or a placebo similar in taste but without supplements, over a period of 12 weeks. After a wash-out period of 12 weeks, the patients received the other test product. The main outcome variable was the levels of glycated hemoglobin (HbA_1c_). Other outcome variables were fasting blood glucose, fructosamine, and lipid parameters.

**Results:**

Thirty-one patients completed the study. HbA_1c_ showed no relevant changes during both treatment periods, nor was there a relevant difference between the two treatments (HbA_1c_: *p*=0.48). The same results were found for fructosamine and fasting glucose (fructosamine: *p*=0.9; fasting glucose: *p*=0.31). In addition, there was no effect on lipid metabolism.

**Conclusion:**

This intervention study does not provide evidence that a cascade-fermented plant-based dietary supplement enriched with a combination of chromium and zinc improves glucose metabolism in patients with type 2 diabetes mellitus under free-living conditions.

Type 2 diabetes mellitus is a major health concern with increasing incidence and prevalence almost worldwide. Up to 30% of the elderly population in Germany is currently treated for diabetes mellitus (1), resulting in an enormous economic burden for the health care system (2). This unfavorable development is a result of the modern lifestyle partly characterized by overnutrition leading to overweight and/or obesity (3). Therefore, dietary habits play an important role in the development and progression of this disease. In this context, dietary supplements have received growing interest as a potential supportive measure to tackle this challenge (4–6).

Chromium is thought to be essential for normal glucose homeostasis (7). Mechanistic studies have shown that chromium improves insulin receptor and post-receptor signaling, resulting in an increased glucose uptake by enhancing the activity of the glucose transporter type 4 (GLUT 4) (8). Severe chromium deficiency can lead to insulin resistance and diabetes, which is reversible by chromium supplementation (9). Some clinical trials with chromium supplementation could show beneficial effects on fasting plasma glucose and/or glycated hemoglobin (HbA_1c_) (10–15). A recent systematic review by Balk et al. (16) concluded that chromium supplementation in diabetic patients is associated with improvements in HbA_1c_ levels by −0.6% and fasting glucose by −1.0 mmol/l. However, in most of these studies, rather high doses of chromium were supplemented (16).

Zinc is a micronutrient that plays multiple roles in a number of cellular and systemic functions (17). Concerning type 2 diabetes mellitus, zinc is an important component for insulin synthesis that functions by stabilizing insulin hexamers and their pancreatic storage (18, 19). Zinc also appears to have an insulinomimetic effect owing to its inhibition of the glycogen-regulating enzyme glycogen synthase kinase 3 (GSK3) (20). In addition, zinc may act as an antioxidant and anti-inflammatory compound (21, 22). Due to hyperglycemia, increased urinary excretion is found in patients with type 2 diabetes resulting in a zinc deficiency (23, 24). Clinical studies with zinc supplementation could show a lowering of HbA_1c_ (25, 26). A meta-analysis by Jayawardena et al. (27) reported mean reductions of HbA_1c_ by 0.54% and fasting blood glucose by 18.13 mg/dl after zinc supplementation.

Additionally, organic acids present in vinegar were shown to exhibit antiglycemic effects (28). However, the mechanism by which the effect is carried out is yet unclear. For example, the acetic acid in vinegar might interfere with disaccharidase activity (29) and/or gastric emptying might be delayed (30). A reduction of postprandial glycemia could be shown, for example, by Östmann et al. (31), who tried out three levels of vinegar (18, 23, 28 mmol), and Johnston et al. (32), who described a ~20% reduction after the ingestion of 10 g of vinegar.

In the present study, we investigated the effect of a dietary product representing a combination of chromium, zinc, and organic acids from the cascade fermentation of vegetable raw materials on glucose metabolism. We used a randomized, placebo-controlled, double-blinded cross-over design to assess the effect of this product on glycemia in well-controlled patients with type 2 diabetes, for 12 weeks under free-living conditions.

## Methods

### Patients

Patients with type 2 diabetes mellitus were screened through advertisements and by personal invitation in general practitioner's practices. Thirty-six non-insulin-dependent diabetic individuals, who met the eligibility criteria, were enrolled. Inclusion criteria were diabetes mellitus type 2 for at least 1 year, treated by diet alone or with oral antidiabetic agents; age between 25 and 73 years; HbA_1c_ >6.5%; and <9.0%, body mass index (BMI) between 25 and 40 kg/m^2^. Exclusion criteria were insulin treatment, uncontrolled arterial hypertension, instable coronary heart disease, or any other severe general diseases, including psychiatric diseases.

### Study design

The study was carried out in the Human Study Center of the Unit of Nutritional Medicine at the Technical University of Munich in Freising-Weihenstephan, Germany. It was registered in the German Clinical Trials Register (DRKS 00003111) and the study protocol was approved by the ethical committee of the Technical University of Munich (Project number: 2866/10). Written informed consent was obtained from all subjects prior to the beginning of the study.

This was a randomized, double-blinded, placebo-controlled trial using a cross-over design with two treatment periods of 12 weeks, each under free-living conditions and separated by a wash-out phase of 12 weeks. The randomization and allocation procedure (blockwise) was prepared by a person not involved in the study. The patients were handed out boxes with their designated number. Sealed envelopes were stored in order to be able to unblind.

### Intervention

The participants received a daily dose of 20 ml of either the test product (Regulat Spezial Diabetic) or a placebo handed out in 20 ml bottles. Ten milliliters had to be taken before breakfast and 10 ml before dinner. Regulat Spezial Diabetic and placebo were produced and provided by Dr. Niedermaier Pharma GmbH (Hohenbrunn, Germany). The test product was prepared by the cascade fermentation of defined vegetables, fruits, and nuts and subsequently enriched with chromium and zinc. The raw ingredients were artichokes, curcuma, dates, peas, millet seeds, figs, coconuts, saffron, celery, soybeans, walnuts, bean sprouts, lemons, and onions. The product provided 100 µg chromium(III)chloride hexahydrate and 15 mg zinc chloride per day. Other ingredients were vitamin C (40 mg/20 ml), niacin (4.8 mg/20 ml), pantothenic acid (1.8 mg/20 ml), pyridoxine hydrochloride (0.51 mg/20 ml), riboflavin (0.42 mg/20 ml), thiamine (0.33 mg/20 ml), folic acid (0.06 mg/20 ml), and cyanocobalamin (0.75 µg/20 ml). The placebo product was designed to have a similar taste and appearance based on orange juice concentrate without any supplements. Compliance was monitored by weighing and counting the number of empty bottles returned by the participants. Adverse effects, changes in body weight, medication, changes in diet or physical activity, and acute illnesses were documented at each visit in week 0, 4, and 12. An additional telephone visit was conducted at week 8. The patients were asked to maintain their normal lifestyle and keep their body weight stable.

### Blood samples

Venous blood samples were drawn in the fasting state (12 h overnight) at baseline and after 4 and 12 weeks in each treatment period. Blood glucose was measured with the HemoCue^®^ system (Großostheim, Germany). Blood samples were centrifuged immediately and stored at −80°C until further analysis. Insulin was determined with a commercially available insulin ELISA kit (DAKO, Denmark) on the ELISA-star platform of Hamilton (Bonaduz, Swiss). All other serum variables were analyzed by a certified laboratory for clinical studies (synlab Labor München Zentrum, Munich, Germany).

### Statistical analysis

Sample size was chosen to detect a difference between treatment and placebo for the primary outcome variable HbA_1c_ with a power of 90% on a two-sided level of significance of 5%. Under the assumption of a true effect size (mean difference divided by standard deviation of the difference) of 0.6 and a drop-out rate of 10%, the sample size resulted in 36 patients.

The method described by Hills and Armitage (33) was used to analyze the data adjusting for a possible period effect. Period effect and treatment by period interaction as well as for differences between the two groups were tested using two sample *t*-test or Mann Whitney U test depending on the distribution of the parameters.

Statistical analyses were conducted with the use of R software, version 2.15.1 (R Foundation for Statistical Computing, Vienna, Austria). All statistical tests were two-sided, with a significance level of 0.05. Data are presented as mean and standard deviation or median and range, where appropriate.

## Results

**Fig. 1:**
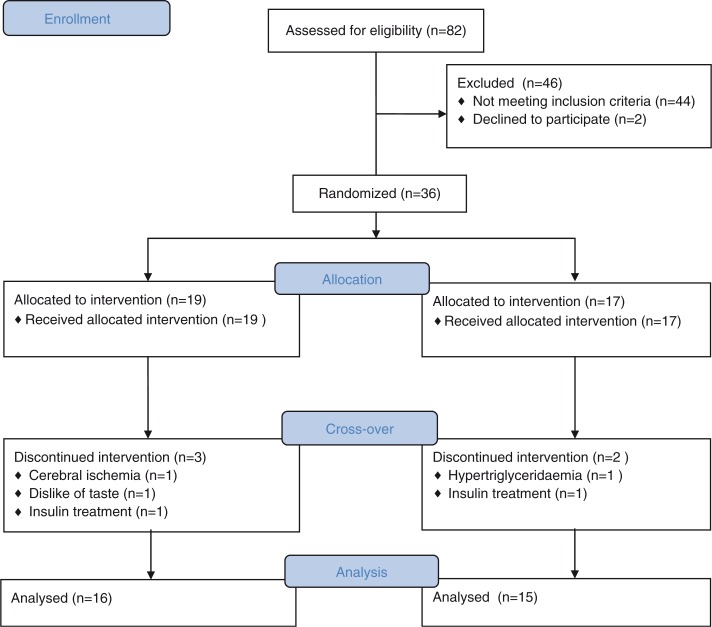
CONSORT 2010 flow diagram of a randomized, placebo-controlled, double-blinded cross-over study of a fermented dietary supplement containing chromium and zinc in type 2 diabetic patients.

The baseline characteristics of the participants at the initiation of the study are presented in Table 1. At the beginning of the study, 50.1% of the patients were treated with metformin, 24.9% with sulfonyl urea, 4.5% with acarbose, 4.5% with dipeptidyl peptidase-4 inhibitor (DPP-4 inhibitor), 11.5% with a combination of DPP-4 inhibitor and metformin, and 4.5% by diet alone. Thirty-one subjects completed the study as presented in the flow diagram (Fig. 1). Reasons for dropping out were elevated triglyceride level (*n*=1), initiation of insulin treatment (*n*=2), cerebral ischemia (*n*=1), and dislike of the taste (*n*=1).

**Table 1:** Baseline characteristics (mean±SD) of the diabetic patients at initiation of the intervention

Characteristic	Test product (*n=*19)	Placebo (*n*=17)
Age (years)	65.0±6.0	61.9±7.6
BMI (kg/m^2^)	30.9±4.0	30.0±5.5
Gender (female/male)	12/7	6/11
Duration of diabetes (years)	12.4±8.0	9.1±6.5
Fasting blood glucose (mmol/l)	141.5±37.3	125.2±19.8
HbA_1c_ (%)	7.3±0.9	7.2±0.6
Fructosamine (µmol/l)	284.3±33.1	267.2±33.1
Cholesterol (mg/dl)	198.0±45.2	206.7±36.7
LDL (mg/dl)	111.8±39.9	117.4±33.8
HDL (mg/dl)	53.8±13.8	48.6±14.0
Triglycerides (mg/dl)	192.9±124.1	202.3±103.4
Uric acid (mg/dl)	5.8±1.6	6.3±1.6
GOT (U/l)	29.6±7.5	27.2±7.7
GPT (U/l)	31.0±11.0	27.2±11.1
Gamma-GT (U/l)	52.0±49.6	41.6±32.0

**Fig. 2:**
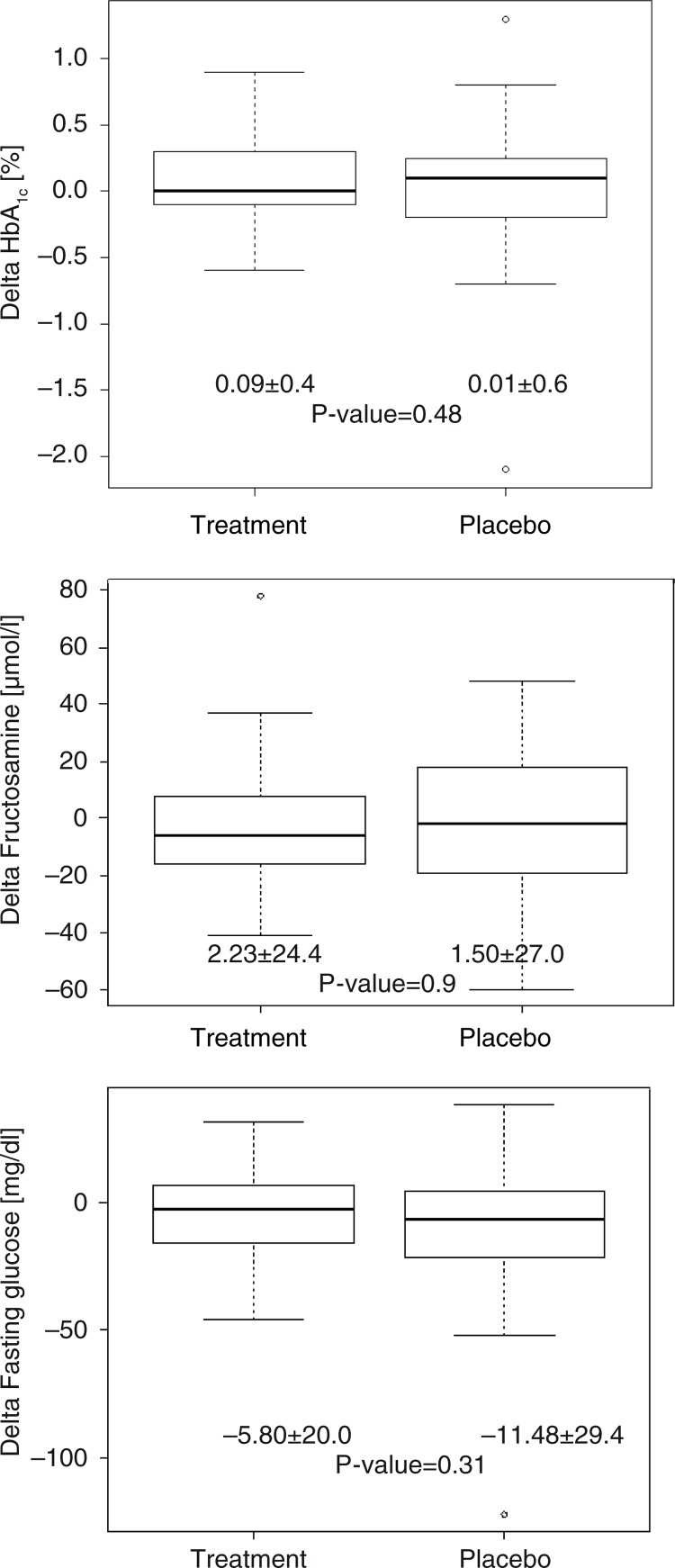
Treatment effect (delta) on HbA_1c_, fructosamine, and fasting glucose for placebo and test product. Data are presented as mean±SD. *N*=31.

HbA_1c_ was the primary outcome variable and was used as a parameter of glycemic control. There was no relevant change in the average values during both treatment periods for the test product (0.09%±0.4) and placebo (0.01%±0.6), respectively. No relevant difference was found between verum and placebo treatment (*p=*0.48) (Fig. 2). We additionally measured fructosamine as another surrogate parameter of glycemia with a shorter elimination rate compared to HbA_1c_. Again, there was no relevant change in the average values for the test product (−2.23 µmol/l±24.4) and placebo (−1.50 µmol/l±27.0), and no difference between the two interventions (*p=*0.9) (Fig. 2). Fasting glucose concentrations also were relevantly affected by neither the test product (−5.80 mg/dl±20.0) and placebo (−11.48 mg/dl±29.4) nor the interventions (*p=*0.31) (Fig. 2).

As other groups could show beneficial changes in blood lipid profiles after chromium or zinc supplementation (12, 34), we also measured the lipid profile as a secondary outcome. However, no relevant differences were observed for total cholesterol (*p=*0.885), HDL (*p=*0.649), LDL (*p=*0.642), and triglycerides (*p=*0.312) at any time point (Table 2). Similar non-relevant results were measured after 4 weeks of treatment (data not shown).

**Table 2:** Treatment effect (Δ) on lipid metabolism, insulin, and zinc levels for the test product and the placebo

	Test product	Placebo
Cholesterol (mg/dl)	−6.5 (−43 to 64)	5.5 (−106 to 108)
HDL cholesterol (mg/dl)	1.0 (−9.0 to 31)	0.5 (−9.0 to 8.0)
LDL cholesterol (mg/dl)	−5.0 (−47 to 71)	3.0 (−92 to 95)
Triglycerides (mg/dl)	−8.5 (−131 to 119)	−11.0 (−128 to 360)
Insulin (U/ml)	−0.45 (−23 to 9.6)	−1.0 (−33 to 15)
Zinc (µg/l)	−4.0 (−128 to 78)	−1.0 (−38 to 29)

Data are presented as median (range). *N*=31.

Both Regulat Spezial Diabetic and a placebo were taken regularly before breakfast and dinner. The test product was well tolerated. During the active intervention period, three patients reported constipation and maldigestion, and two patients mentioned heartache or retrosternal burning after swallowing. During placebo treatment, four patients reported gastrointestinal symptoms, e.g. diarrhea, constipation; and two patients mentioned retrosternal pain or burning after taking the solution. One patient had pustules both under the placebo and the test product. Body weight, dietary habits, and physical activity remained unchanged during both intervention periods (data not shown).

## Discussion

Dietary factors play a pivotal role in the treatment of type 2 diabetes and have acute and long-term effects on blood glucose excursions and finally on HbA_1c_. Certain nutritional supplements like chromium, zinc, and vinegar have been shown to support the control of hyperglycemia in patients with type 2 diabetes suggested by data from different sources, including animal experiments and human studies (8, 10, 12, 25, 26, 34).

This fermented extract of fruits, nuts, and vegetables has been promoted as a natural product with a glucose-lowering activity, although not yet formally proven. Therefore, the initial concept was to combine different glucose-lowering dietary components, namely chromium, zinc, and organic acids. This was achieved by adding chromium and zinc to the fermented extract. As to the used doses of the micronutrients, the recommendations of the German Nutrition Society (DGE) (35) were followed. These allowed doses were moderate compared to those of other studies, but as this study aimed to have a combined glucose-lowering effect, moderate doses were chosen.

The strategy for chromium supplementation is controversially discussed with regard to the daily dosage. The dosages in studies reporting positive effects on glycemic parameters ranged from 100 up to 1,000 µg chromium daily. However, there was no clear dose-effect relationship (16). Our test product provided 100 µg/d and was administered as chromium(III)chloride hexahydrate. This supplementation seems justified to compensate for the higher loss of chromium (III) via the urine reported for patients with type 2 diabetes (24, 36), but there was no effect on any metabolic parameter measured in our study.

Regarding zinc supplementation, clinical studies showed positive effects on glycemic control like the one by Al-Maroof and Al-Sharbatti (25) and Gunasekara et al. (26), who administered 30 mg and 22 mg zinc sulfate, respectively. There are also studies with a daily dose of 30 mg zinc gluconate administered for 6 months, reporting no effect on HbA_1c_ (37, 38). We used an amount of 15 mg zinc chloride per day but could not detect any significant effect.

The test product also contained organic acids derived from cascade fermentation of various fruits, nuts, and vegetables, which resulted in a vinegar-like composition. Johnston et al. (32) were able to show a reduction of postprandial glycemia of ~20% by ingesting 2 tablespoons of vinegar per day. We did not observe any effect on blood glucose levels in our study. This discrepancy may be explained by differences in the composition of organic acids. The main organic acid in the test product was lactic acid in contrast to acetic acid in vinegar. It was hypothesized by Ogawa et al. (29) that acetic acid, but not organic acids such as lactic and citric acid, inhibit disaccharidase activity in the small intestine.

The combination of different glucose-lowering dietary components in moderate doses did not show any significant positive effects on glucose metabolism. The doses might have been too low. Thus, we cannot fully exclude the possibility that higher doses of chromium, zinc, and organic acids may be necessary to elicit significant effects on glucose and lipid metabolism in subjects with type 2 diabetes.

The strength of this intervention is the study design itself. Cross-over studies are advantageous for studies of chronic diseases like type 2 diabetes mellitus and the influence of confounding covariates is reduced because each cross-over patient serves as his or her own control.

A limitation of this trial is that we were not able to show any positive effects on glucose and lipid metabolism, even though a power calculation had been performed and the study was carefully conducted according to the protocol.

## Conclusion

Taken together, our study does not provide evidence that a product based on cascade-fermented fruits, nuts, and vegetables combined with chromium and zinc supplementation at moderate doses has a beneficial effect on glucose and lipid metabolism in subjects with type 2 diabetes.
